# Comparing knowledge, attitudes and practices regarding COVID-19 amongst Cameroonians living in urban versus rural areas

**DOI:** 10.11604/pamj.2021.38.234.25964

**Published:** 2021-03-04

**Authors:** Atabong Emmanuel Njingu, Fombo Enjeh Jabbossung, Tambe Emilia Ndip-Agbor, Ankwatia Guisilla Dedino

**Affiliations:** 1 Faculty of Health Sciences, University of Buea, Buea, Cameroon,; 2 Kalfou Integrated Health Center, Kalfou, Cameroon,; 3 Saint John of God Health Center, Mamfe, Cameroon,; 4 Faculty of Health Sciences, University of Bamenda, Bamenda, Cameroon,; 5 Faculty of Medicine and Biomedical Sciences, Yaoundé, Cameroon,; 6 Muea Medicalized Health Center, Muea, Cameroon

**Keywords:** COVID-19, knowledge, attitudes, practices, rural areas, urban areas

## Abstract

**Introduction:**

adherence to preventive measures to curb the spread of COVID-19 depends on the people´s knowledge, attitudes and practices (KAP) towards COVID-19. Living in rural areas may be associated with poor KAP towards COVID-19. This study compares the KAP regarding COVID-19 of people living in rural and urban areas in Cameroon.

**Methods:**

this was a comparative cross-sectional study, using data obtained through an online survey of 1,345 Cameroonians amongst which were 828 urban and 517 rural dwellers. The survey questionnaire consisted of; demographic characteristics, 10 questions on Knowledge, 4 on attitudes and 3 on practices. Data was analyzed using SPSS version 25.

**Results:**

overall, about two-thirds of participants had correct knowledge of COVID-19. The mean knowledge score for urban dwellers was about twice that of rural dwellers (15.77 ± 5.25 vs 8.86 ± 7.24 respectively, p < 0.001). Furthermore, when compared to people who live in urban areas, rural inhabitants are less optimistic about COVID-19 pandemic in Cameroon (OR = 3.43, P<0.001), less likely to accept a trial vaccine for COVID-19 (OR = 1.14, P<0.05), less likely to avoid going to crowded places (OR = 7.42, P<0.01), less likely to wear face mask outdoor (OR = 11.84, P<0,001), and less likely to practice hand hygiene (OR = 1.13, P<0.05).

**Conclusion:**

our findings suggest a big gap in COVID-19 related knowledge, attitudes, and practices between rural and urban inhabitants in Cameroon. This highlights the need for increase sensitization of Cameroonians, especially rural dwellers on COVID-19 related knowledge, attitudes and appropriate practices.

## Introduction

Coronavirus disease 2019 (COVID-19) is an emerging illness caused by a novel form of coronavirus called; severe acute respiratory syndrome coronavirus 2 (SARS-CoV-2) [1]. The main clinical symptoms of COVID-19 are; cough, fever, dyspnea, fatigue, myalgia. Morbidity due to COVID-19 ranges from mild respiratory symptoms to severe acute respiratory distress, septic shock, severe metabolic and hemostasis disorders that may lead to death [2-4]. Older people (people over 60 years), and those with comorbidities such as; cardiovascular diseases, diabetes, chronic lung diseases, and cancer are at higher risk of getting severe COVID-19 disease [5]. Since its inception in December 2019 in Wuhan city, China, COVID-19 has spread to almost all countries in the world. The World Health Organization (WHO) declared COVID-19 a pandemic on the 11^th^ March 2020 [6]. Recent statistics show that there are over 22 million confirmed cases of COVID-19 worldwide and about 1 in every 5 infected persons develop difficulty in breathing that requires hospital care. The crude mortality ratio due to COVID-19 is between 3-4% [5,7,8]. In Cameroon, there are over 18,700 confirmed cases and over 400 recorded deaths due to COVID-19 [9]. The rapid spread of this virus and its global effect requires collaborative efforts of all countries and individuals to control its spread [10]. The success of the measures put in place by government to curb the spread of COVID-19 is hugely dependent on public adherence to these measures. In accordance with the knowledge, attitude and practices (KAP) theory and lessons learned from SARS outbreak in 2003 [11-13], public adherence is in turn affected by their KAP towards the disease. Assessing public KAP helps in identifying gaps and strengthening ongoing prevention efforts. This study was assessment of the difference in the knowledge, attitude and practices of Cameroonians living in rural and urban areas of the country.

## Methods

**Study design, period and setting:** this was a comparative cross-sectional study conducted among the general population of Cameroon from July 10 - 24^th^ 2020. Given the social distancing (physical distancing) measures and movement restrictions, data were collected using a pretested online questionnaire, designed using Google Forms. The questionnaire was available in English and in French. A link to the survey was distributed to potential respondents via Twitter, WhatsApp and Facebook groups of authors and collaborators.

**Population and sampling:** the study population was made up of males and females, aged 18 years and above, residing in Cameroon. All those who consented to the study were allowed access to the rest of the online questionnaire. A target sample size of 1,083 was obtained using a sample size calculator [14]. A margin of error of ±5%, confidence level of 99%, a 50% response distribution, and a population of 25,220,000 people was used, based on most recent population census in Cameroon [15]. This study had 1,345 participants.

**Study procedures and variables:** data was collected using an online questionnaire adapted from other COVID-19 studies [2,6,10,16]. To obtain knowledge score, respondents were asked to respond to questions about COVID-19, symptoms, mode of transmission, and treatment. Responses were either true, false or “I don't know”. Incorrect/uncertain responses were given a score of zero, and correct answers were assigned a score of one. The total score for knowledge ranged from 0-21, participants who scored 13 and above were considered to have good knowledge of COVID-19, knowledge scores of less 13 were considered as poor. The link to the online questionnaire was shared through social media platforms (“WhatsApp” groups. Twitter, Facebook) as an open invitation for individuals that are 18 years and above to click on the link, consent to the study, then fill the rest of the online questionnaire. The questionnaire consisted of; socio-demographic data, participants´ awareness of COVID-19, preventive measures, clinical manifestations, treatment, attitudes toward COVID-19, and respondents' practices regarding COVID-19. Study population was divided into rural and urban base on preexisting classification of towns in Cameroon by World Bank and Cameroon encyclopedia. With this classification, areas with less than 1500 inhabitants per square kilometer (km^2^) were considered rural and areas with inhabitants above 1500/km^2^ were considered urban [17,18]. In the questionnaire, respondents were asked to state precisely where they live. Their responses were coded into rural and urban base on above mentioned classification, people living in areas not classified by the existing classification were assigned to rural or urban base on the population density of the area they live in.

**Data management and data analysis:** data were analyzed using the Statistical Package for the Social Sciences (SPSS), version 25. Descriptive analyses were made. Chi-square tests, independent samples t-tests and one-way analysis of variance (ANOVA) were utilized to determine the differences between groups for selected demographic variables. Multivariable linear regression analysis was conducted to identify factors associated with knowledge score. The statistical significance level was set at p < 0.05. Respondents were asked to respond to knowledge items as either true, false or “I don't know”. Incorrect/uncertain responses were given a score of zero, and correct answers were assigned a score of one. The total score for knowledge ranged from 0-21, with higher scores indicating better knowledge of COVID-19. To assess attitudes and practices, scores were calculated based on the respondents' answers to each attitudinal statement, 1 = very fearful, 2 = fearful, 3 = fearful but optimistic, 4 = optimistic, and 5 = very optimistic. Participants were also asked yes/no questions on whether they had avoided going to crowded places, if they trust the information they get about COVID-19, wore face mask when leaving home, and practiced of hand hygiene. The Likert scales were assessed for internal reliability, using Cronbach's α. The Cronbach's α coefficient for the knowledge, attitude, and practice questionnaires were; 0.75, 0.69, and 0.81 respectively, indicating acceptable internal consistency [19].

**Ethical considerations:** ethical approval was obtained from the Institutional Review Board of the Faculty of Health Sciences of the University of Buea (approval number: 2020/1260/UB/SG/IRB/FHS). Participants also gave consent to willingly participate in the survey by clicking the ‘Accept´ button and were then directed to complete the questionnaire.

## Results

**Socio-demographic characteristics of study participants and COVID-19 knowledge score:** a total of 1345 participants completed the questionnaire. Of these total, 828 (61.56%) persons were residing in urban areas and 517 (38.44%) in rural areas, 819 (60.89%) were females and most (46.17%) were in the 40 - 49 age group. Other demographic characteristics are detailed in Table 1. Overall, the mean COVID-19 knowledge score was 13.12 (SD = 6.96, range: 3-21) suggesting an overall 62.48% (13.12/21*100) accuracy rate for the knowledge test. The mean knowledge score for urban inhabitants was significantly higher than that of rural dwellers (15.77 ± 5.25 vs 8.86 ± 7.24 respectively, p < 0.001). The mean knowledge score also significantly differed across gender, employment sector and level of education (Table 1). Multiple linear regression analysis showed that rural residence (vs. urban, β: 0.483, P<0.01), female gender (vs. male, β: 0.078, P<0.01), age group of 50+ years (vs. 20-39 years, β: -0.085, P<0.01), educational level of secondary or lower (vs. university/higher, β: -0.163~ -0.205, P<0.001), and employment sector of transport (β: -0.286, P=0.040) and unemployed (β: -0.221, P<0.001) (vs administration) were significantly associated with lower knowledge score.

**Table 1:** demographic characteristics of participants and knowledge score of COVID-19

Characteristics		Number of participants (%)	Mean knowledge score ± standard deviation	t/F	P value
Gender	Male	526 (39.11)	13.79 ± 6.76		
	Female	819 (60.89)	12.68 ± 7.05	8.986	0.004
Age group	<20	25 (1.86)	11.68 ± 4.91		
	20 - 39	604 (44.91)	13.49 ± 6.68		
	40 - 49	621 (46.17)	13.39 ± 7.21		
	50+	95 (7.06)	9.32 ± 6.37	11.22	0.067
Employment sector	Education	286 (21.26)	16.41 ± 5.02		
	Farming	278 (20.67)	7.64 ± 6.88		
	Health care	195 (14.50)	16.99 ± 3.24		
	Grocery/supermarket	179 (13.31)	14.06 ± 7.06		
	Administration	130 (9.67}	17.07 ± 3.12		
	Others	118 (8.77)	14.35 ± 4.58		
	Unemployed	108 (8.03)	10.65 ± 6.08		
	Transportation	51 (3.79)	8.51 ± 7.96	261.36	<0.001
Place of residence	Urban	828 (61.56)	15.77 ± 5.25		
	Rural	517 (38.44)	8.86 ± 7.24	247.92	0.000
Education	Secondary	690 (51.30)	10.00 ± 7.41		
	University/Higher	640 (47.58)	16.62 ± 4.23		
	Primary	15 (1.12)	6.87 ± 6.69	198.37	0.048

"Others" included; janitors, soldiers, seamstress, artist, accountants, construction workers

**Figure 1:**
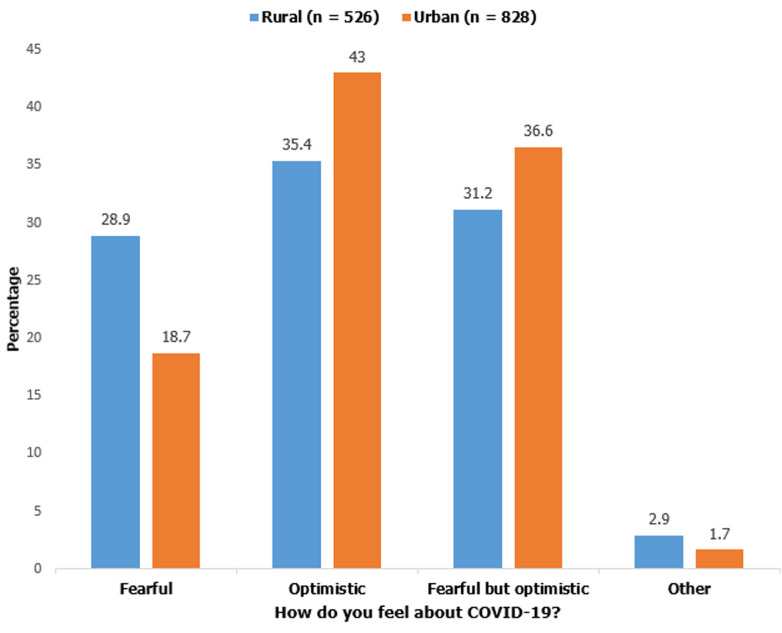
attitude towards COVID-19 and place residence

**Attitudes/perceptions of the study participants towards COVID-19:** study participants were asked how they feel about COVID-19; 152 (28.9%) of rural dwellers vs 155 (18.7%) of urban inhabitants (p<0.01) reported being fearful of COVID-19 (Figure 1). Participants were also asked if they trust the information they get about COVID-19, if they can accept a trial vaccine/treatment and if they avoided going to the hospital due to fear of COVID-19 suspicion. The results in Table 2 shows significant variation in responses across different demographic variables.

**Table 2:** attitudes towards COVID-19 and demographic characteristics

Characteristics		Attitude, n (%)							
		Do you think you can fully protect yourself against COVID-19		Avoid going to the hospital due to fear of COVID-19 suspicion		Will you accept a trial vaccine		Do you trust the information you get about COVID-19	
		Yes	No	Yes	No	Yes	No	Yes	No
Gender	Male	213 (40.49)	313 (59.51)	295 (56.08)	231 (43.92)	145 (27.57)	381 (72.43)	157 (29.85)	369 (70.15)
	Female	265 (32.36)	554** (67.64)	512 (62.52)	307 * (37.48)	120 (14.65)	699** (85.35)	287 (35.04)	532 (64.96)
Age group	<20	21 (84.00)	4 (16.00)	9 (36.00)	16 (64.00)	0 (0.00)	25 (100)	11 (44.00)	14 (56.00)
	20 - 39	247 (40.89)	357 (59.11)	338 (55.96)	266 (44.04)	134 (22.19)	470 (77.81)	202 (33.44)	402 (66.56)
	40 - 49	167 (26.89)	454 (73.11)	398 (64.09)	223 (35.91)	128 (20.61)	493 (79.39)	174 (28.02)	447 (71.98)
	50+	43 (45.26)	52 *** (54.74)	62 (65.26)	33 ** (34.74)	3 (3.16)	92 *** (96.84)	57 (60.00)	38*** (40.00)
Employment sector	Education	205 (71.68)	81 (28.32)	169 (59.09)	117 (40.91)	44 (15.38)	242 (84.62)	52 (18.18)	234 (81.82)
	Farming	150 (39.68)	228 (60.32)	296 (78.31)	82 (21.69)	60 (15.87)	318 (84.13)	243 (64.29)	135 (35.71)
	Health care	49 (25.13)	146 (74.87)	1 (0.51)	194 (99.49)	62 (31.79)	133 (68.21)	143 (73.33)	52 (26.67)
	Grocery/ supermarket	42 (23.46)	137 (76.54)	123 (68.72)	56 (31.28)	17 (9.50)	162 (90.50)	58 (32.40)	121 (67.60)
	Others	16 (25.40)	47 (74.60)	28 (44.44)	35 (55.56)	5 (7.94)	58 (92.06)	5 (7.94)	58 (92.06)
	Unemployed	27 (42.86)	36 (57.14)	19 (30.16)	44 (69.84)	7 (11.11)	56 (88.89)	45 (71.43)	18 (28.57)
	Administration	81 (62.31)	49 (37.69)	80 (61.54)	50 (38.46)	63 (48.46)	67 (51.54)	11 (8.46)	119 (91.54)
	Transportation	32 (62.75)	19 *** (37.25)	40 (78.43)	11 ** (21.57)	7 (13.73)	44 *** (86.27)	28 (54.90)	23 *** (45.10)
Place of residence	Rural	176 (34.04)	341 (65.96)	340 (65.76)	177 (34.24)	95 (18.38)	422 (81.62)	286 (55.32)	231 (44.68)
	Urban	302 (36.47)	526 (63.53)	467 (56.40)	361 (43.60)	170 (20.53)	658 (79.67)	208 (25.12)	620*** (74.88)
Education	Primary	9 (60.00)	6 (40.00)	9 (60.00)	6 (40.00)	0 (0.00)	15 (100.0)	11 (73.33)	4 (26.67)
	Secondary	240 (34.78)	450 (65.22)	480 (69.57)	210 (30.43)	97 (85.94)	593 (85.94)	326 (47.250	364 (52.75)
	University/ Higher	229 (35.78)	411 (64.21)	318 (49.69)	322*** (50.31)	168 (26.25)	472*** (73.75)	157 (24.53)	483 (75.47)
Knowledge score (t-test)		13.21 (2.71)	13.50** (1.82)	12.91 (2.90)	13.02* (2.70)	14.23 (1.83)	13.13** (2.91)	13.51 (1.82)	13.70** (1.22)

"Others" included; janitors, soldiers, seamstress, artist, accountants, construction workers *P<0.05, **P<.01, ***P<0.001. P values assessed the significance in the different results across demographic variables

**Figure 2:**
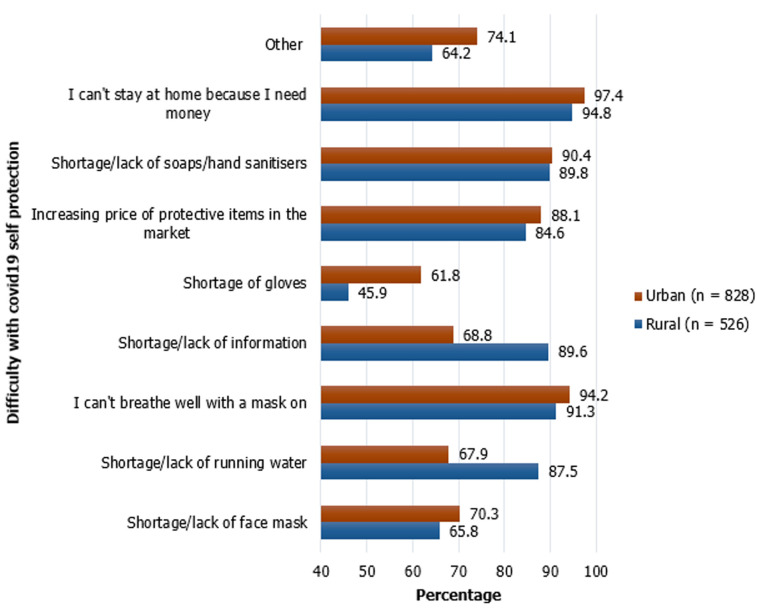
difficulty with COVID-19 self-protection and place of residence

**Practices of the study participants towards COVID-19:** most (n=1,122, 83.42%) of the study participants avoided going to crowded places in order to prevent spread of COVID-19. However, a significantly higher percentage of urban dwellers avoided crowded places when compared to rural dwellers (89.9% vs 73.1%, p<0.01). In addition, a significantly higher percentage of urban inhabitants wore face mask outdoor and practiced hand hygiene (Table 3). Variation in practices were also seen across gender, employment sector, level of education and age groups (Table 3). Study participants were also asked what difficulty they have with respecting preventive measures against COVID-19; more than 95% of respondents will not stay at home because they need money, others reported lack/shortage of face mask, difficulty in breathing with face mask on and shortage/lack of information (Figure 2).

**Table 3:** demographic characteristics of participants and practices toward COVID-19

Characteristics		Practices, n (%)					
		Avoid going to crowded places		wear face mask before leaving the house		Regular hand hygiene	
		Yes	No	Yes	No	Yes	No
Gender	Male	437 (83)	89 (17)	509 (96.8)	17 (3.2)	442 (83.9)	84 (16.1)
	Female	704 (86.0)	115 (14.0)	794 (97.3)	25 (2.7)	745 (90.7)	74*** (1.3)
Age group	<20	19 (74.9)	6 (25.1)	24 (98.1)	1 (1.9)	22 (90.3)	3 (9.7)
	20 - 39	501 (83.7)	103 (16.3)	580 (96.3)	24 (3.7)	507 (84.2)	97 (15.8)
	40 - 49	528 (84.9)	93 (15.1)	602 (97.2)	19 (2.8)	540 (87.4)	81 (12.6)
	50+	72 (76.2)	23 ** (23.8)	92 (96.6)	3 (3.4)	77 (81.2)	18 *** (18.8)
Employment sector	Education	237 (82.8)	49 (17.2)	277 (97.1)	9 (2.9)	252 (88.2)	34 (11.8)
	Farming	234 (84.4)	44 (15.6)	264 (94.7)	14 (7.3)	239 (86.0)	39 (14.0)
	Health care	162 (82.9)	33 (17.1)	174 (88.9)	21 (10.1)	177 (90.7)	18 (9.3)
	Grocery/ supermarket	140 (77.6)	39 (23.4)	168 (94.4)	11 (5.6)	161 (89.8)	18 (10.2)
	Others	100 (84.8)	18 (15.2)	112 (95.1)	6 (4.9)	105 (89.1)	13 (10.9)
	Unemployed	87 (81.3)	21 (18.7)	105 (97.2)	3 (2.8)	84 (78.0)	24 (22.0)
	Administration	107 (82.4)	23 (17.6)	125 (96.2)	5 (3.8)	111 (84.9)	19 (15.1)
	Transportation	44 (85.9)	7 (14.1)	46 (90.8)	5 (9.2)	44 (85.8)	7 ** (14.2)
Place of residence	Rural	377 (73.1)	140 (26.9)	445 (86.1)	72 (13.9)	439 (85.3)	78 (14.7)
	Urban	745 (89.8)	83 ** (10.2)	803 (97.2)	25*** (2.8)	762 (92.1)	66* (17.9)
Education	Primary	12 (80.8)	3 (19.2)	13 (86.7)	2 (13.3)	14 (87.1)	1 (6.7)
	Secondary	538 (78.0)	152 (22.0)	629 (91.2)	61 (8.8)	593 (85.9)	97 (14.1)
	University/ Higher	538 (84.0)	102 *** (16)	557 (87.0)	83* (13.0)	563 (88.3)	77 (11.7)
Knowledge score (t-test)		13.1 (2.7)	13.6*** (1.6)	13.9 (1.6)	12.5*** (3.4)	13.1 (2.7)	13.1 (2.7)

"Others" included; janitors, soldiers, seamstress, artist, accountants, construction workers *P<0.05, **P<.01, ***P<0.001. P values assessed the significance in the different results across demographic variables

## Discussion

COVID-19 is a relatively new infectious disease of significant global interest. The devastating effects of COVID-19 is not just limited to health but also on the global economy. Without a vaccine, practicing preventive measures remain the mainstay in controlling the spread of the virus. Public adherence to preventive measures hugely rely on their knowledge, attitudes and practices (KAP) towards the disease. Thus this study aimed to compare the KAP of Cameroonians leaving rural and urban parts of the country towards COVID-19. The mean knowledge score of Cameroonians regarding COVID-19 was good (13.12) and the overall correct answer rate was 62.48%. These scores however do not reflect the different groups of participants. The mean knowledge score of urban dwellers was about twice that of rural dwellers (15.77 vs 8.86 respectively, p<0.001). This significantly higher level of COVID-19 knowledge amongst urban inhabitants found in this study is similar to findings by Akalu *et al*. in Ethopia [2], the better knowledge score amongst urban dwellers is thought to be due to the poor access to electricity and internet in rural areas of Cameroon [20], as a result, rural inhabitants have limited access to COVID-19 related information shared on the internet and other media that have been shown to have positive effect on improving knowledge [2]. In addition, the poor security situation in the rural areas in the extreme North, the Southwest and Northwest Regions of Cameroon makes access to these areas by health promotion team difficult. The overall correct response rate for COVID-19 knowledge questions was good for this study. Nevertheless, the value (62.48%) is smaller than the results for many other studies [10,21]. The difference could be due to the fact that, our study was carried in both rural and urban areas unlike other studies [2,6,10,22] carried out in urban settings and among people with high educational standards. Similar to other studies, significant variation in knowledge score was observed across gender, employment sector and level of education.

Majority of the study participants in this study were either optimistic or fearful but optimistic. A significantly higher percentage (79.6%) of urban dwellers were optimistic about COVID-19 when compared with people leaving in rural areas (66.6%). As reported by other studies in Asia and Africa [10,21,22], higher knowledge score was strongly associated with positive attitudes towards COVID-19. In this study, urban inhabitants had higher mean COVID-19 knowledge score which could account for their higher level of optimism. Despite this optimism, 65.8% of people living in rural areas and 56.4% of people living urban areas (p<0.001) avoided going to the hospital due to fear of being suspected of having COVID-19. More so, only 20.5% of urban inhabitants and 18.4% of rural inhabitants will accept a trial vaccine against COVID-19. In addition, 25.1% of urban and 55.3% of rural dwellers do not trust the information they get about COVID-19. Social stigma associated with COVID-19 infection [13,23], plus the stress, anxiety and depression associated with isolation/quarantine [24] could be the reasons for people being afraid of going to the hospital in order to avoid suspicion of being infected with COVID-19. Since the proposal in April 2020 by some French doctors for COVID-19 vaccines to be tested in Africa [25], there have been widespread condemnation and skepticism about COVID-19 vaccine in Africa, with reported protest against the use of the vaccine in some African countries [26].

The suggestion was seen as a racist move with Africans being treated as inferior human beings [26]. This view of the vaccine could explain why it is hated in Africa. The thought of COVID-19 pandemic being politicized, with reported misuse of its funds by some media [27] and poor knowledge about the pandemic, may suggest why most rural dwellers doubt the information they get about COVID-19. Many studies conducted in Asian countries have indicated positive attitudes towards COVID-19 among the general population and healthcare workers [10,21,22]. Differences in measurement and scoring systems do not make it possible for accurate comparisons of attitudes across these studies. Despite the poor knowledge and negatives attitudes towards COVID-19 by most rural dwellers and some urban inhabitants, our results show that, there are significant differences between the two groups. Most study participants (both rural and urban inhabitants) carried out good practices towards COVID-19, more than 80% of all participants reported wearing mask outdoor, practiced regular hand hygiene and avoided crowded places. High levels of safe practices towards COVID-19 were also reported by other studies [10,21,22]. Strict government regulations and the use of forces of law and order to ensure these preventive measures are respected were suggested as reasons for the high levels of positive practices towards COVID-19. Similar to other studies [10,22], higher COVID-19 knowledge score was found to be associated with higher likelihood of safe practices towards COVID-19.

**Study limitations:** sampling for this study was by convenience sampling through the networks of the authors and shared through different social media sites. Hence, there is a chance of bias as some underprivileged people may not have been able to participate in the study. Another limitation of this study is the possibility of participants giving socially desirable answers. For this study with self-reported data, it is possible that respondents may have answered attitude and practice questions positively/negatively based on what they perceived to be expected of them.

## Conclusion

Our findings suggest that, urban inhabitants in Cameroon are about twice as knowledgeable about COVID-19 as their rural counterparts. Though worse in rural settings, there is a general negative behavior by Cameroonians of both rural and urban settings towards COVID-19 and the strictly implemented preventive measures by the government have helped most people to carry out safe practices. This survey brings out the need for targeted health education interventions directed towards people living in rural areas. Furthermore, COVID-19 information shared to the public should also be adapted to meet people of different educational backgrounds, age groups and sectors of employment.

### What is known about this topic

- About 66% of Cameroonians living in Buea have good or intermediate knowledge about COVID-19;
- Level of COVID-19 knowledge is strongly associated with attitudes and practices towards the disease;
- Most people display positive attitudes towards COVID-19.

### What this study adds

- This study is the first in Cameroon to the best of the researchers´ knowledge to compare KAP amongst Cameroonians living in rural and urban settings;
- This study is also the first to assess participants´ attitude towards the hospital and willingness to accept a trial vaccine during this pandemic;
- Our study shows that the level of COVID-19 knowledge varies grossly amongst people living in urban and those living in rural areas in Cameroon.
